# The Impact of Training on Electronic Health Records Related Knowledge, Practical Competencies, and Staff Satisfaction: A Pre-Post Intervention Study Among Wellness Center Providers in a Primary Health-Care Facility

**DOI:** 10.2147/JMDH.S414200

**Published:** 2023-06-02

**Authors:** Sarah Musa, Ismail Dergaa, Rawia Al Shekh Yasin, Rajvir Singh

**Affiliations:** 1 Department of Preventative Health, Primary Health Care Corporation (PHCC), Doha, Qatar; 2 Department of Quality & Patient Safety, Primary Health Care Corporation (PHCC), Doha, Qatar; 3 Department of Adult Cardiology, Heart Hospital, Hamad Medical Corporation (HMC), Doha, Qatar

**Keywords:** Electronic health record, health care, health care providers, health care quality, health care safety, health informatics, health information technology

## Abstract

**Background:**

The transition to electronic health records (EHR) has improved the quality of health-care delivery and patient safety. However, poor usability and incongruent workflow may impose a significant burden on documentation and time management, resulting in staff burnout. We aimed to (i) evaluate the effectiveness of personalized EHR training on wellness providers’ knowledge and practical competencies, and (ii) assess staff satisfaction regarding the EHR usage post-training.

**Methodology:**

An interventional study was conducted between July 15, 2021, and March 1, 2022, among 14 wellness staff (age: 38 ± 3.9 years; 7 males, 7 females) in the Wellness Center-Rawdat Al-Khail Health Center. Six months of blended training was delivered. The impact of training was assessed using a pre-post survey on the knowledge and practical competencies related to EHR usage. Staff satisfaction was assessed post-training.

**Results:**

Majority of respondents had improvement in identifying the advantages of EHR: improve confidentiality of care (pre = 35.7% vs post = 100%, p = 0.001), reduce medical errors (pre = 35.7% vs post = 85.7%, p = 0.02), improve quality of health care (pre = 35.7% vs post = 100%, p = 0.001), and reduce wait time (pre = 42.9% vs post = 85.7%, p = 0.03). Time performing these tasks by massage therapists/receptionists was reduced: viewing/editing ambulatory organizer (pre = 20±0 s vs post = 10±0 s), access PM office (pre = 155±136 s vs post = 10±0 s), selection/access patient chart (pre = 75±30 s vs post = 30±20 s), check-in/out (pre = 120±0 s vs post = 60±0 s), and view/edit massage form (pre = 135±75.5 s vs post = 60±0 s). For gym instructors, time to access ambulatory organizer (pre = 30±0 s vs post = 10±0 s), view/edit the gym form (pre = 101±57 s vs post = 71±36 s), view patients’ clinical data (pre = 60±70 s vs post = 10±3 s), and place referral orders (pre = 197±144 vs post = 82±23 s) was reduced. A mean percentage score of 65.4±38.7 indicated very good staff satisfaction.

**Conclusion:**

This tailored, hands-on training has been well received and effectively improved wellness staff knowledge, competencies, and satisfaction relative to EHR functionalities.

## Background

Electronic Health Records (EHR) are variably defined among different health organizations conditional to their specific scopes and intended purposes.^1^ Principally, EHR are referred to as systematic collection and maintaining of patients’ health information capable of improving the quality of health-care delivery system and patients’ safety. Successful shifting from paper-based into EHR system can be challenging, however, it has proven to enhance the efficiency in care delivery through data exchange and better decision support.^1^,^2^

EHR embrace several functionalities such as viewing health information, placing orders, scheduling appointments, and billing. They are, therefore, regarded as a key tool to reduce care costs, reduce medical errors, enhance continuity of care, surveillance, and adherence to health management guidelines.^3^,^4^ EHR also have the potential to overcome limitations related to paper-based records such as storage problems, written errors, and lost or misfiled charts that could threaten the privacy of the patients.^3^,^4^ EHR have been shown to improve quality measures in relation to the prevention, screening, and management of chronic diseases.^5^ A randomized controlled trial of 21 practices demonstrated a reduction in blood pressure among patients with hypertension who received screening and advice on high-risk drinking, alcohol abuse, or alcohol dependence through an EHR intervention.^6^ Furthermore, increasing the visibility of laboratory data means providers can deliver excellent care at a lower cost, by reducing errors and test utilization.^7^ Numerous resources and tools, such as assessments for drug interactions, Framingham calculators, and body mass index (BMI) calculators, can help providers instantly monitor health data over time and intervene earlier. Data suggest that patients tend to give a higher rating of quality care if their physicians use EHR to maintain their health records.^8^

Despite the growing literature on the benefits of EHR, unintended consequences and poor usability have been adversely correlated with this technology.^9^ These include changes in workflow, temporary loss of productivity, privacy and security concerns, negative emotions (burnout/dissatisfaction), overdependence on technology, and financial issues related to EHR adoption, implementation, and monitoring.^9^,^10^ Increased documentation time may be reflected on patient-provider communication, hence accuracy of medical information, and poses a barrier to effective implementation of EHR.^9^,^10^ However, most of the previous studies have examined the impact of the EHR system on physicians and nurses with a limited number focusing on paramedical staff such as gym instructors and massage therapists within a fitness facility.

In order to optimize the integration of EHR within the routine clinical workflow, early phase planning should be considered. Researchers have found an association between the use of EHR and increased medical errors due to poorly designed system interfaces or lack of end-user training.^11^ For example, physicians who did not receive adequate training were nearly four times more likely to report that their EHR did not enable them to deliver quality care.^12^ However, most health-care organizations provide initial basic training that focuses mainly on mastering software functionalities rather than tailored discrete sessions and often taught as a standard Human Resources onboarding component instead of a competency requirement. Studies have found that up to 94.6% of respondents claimed their ability to use EHR could be improved,^13^ while 75% felt the need for additional training five years after the EHR implementation.^14^ These studies indicated that by giving health-care future and current professionals hands-on, case-based training, learning and self-efficacy increase, especially when conducted within the actual work environment.^15^,^16^

In the context of preventative health and lifestyle fields, the effective use of EHR may help to maintain the recommended levels of physical activity (PA), improve progress, and adherence to exercise programs.^17^ Consequently, EHR exerts an influence on metabolic, hemodynamic, body composition, epigenetic, and functional risk factors related to non-communicable diseases (NCDs).^18^ In Southern California, the Kasier Performance study indicated that the integration of patients’ physical activity data into their EHR has shown considerable promise for improving patient treatment and care quality.^19^,^20^ The study revealed that two-thirds of those who had the exercise vital signs in their records were meeting the national guidelines for PA.^19^

In Qatar, most of the health services at the Primary Health Care Corporation (PHCC) were transitioned into the EHR system by 2014. Wellness Centers, which are under the Preventative Health department, effectively went live in August 2022 at five HCs, with an exception of Rawdat Al-Khail Health Center (RAK) HC, being as a designated COVID-19 facility. Wellness services within RAK HC were suspended and alternatively wellness providers were assigned duties relative to COVID-19.

From 2017, the operation of Wellness Centers including setting appointments, referrals, assessment, and form-filling such as Physical Activity Readiness Questionnaire (PAR-Q), outcome gym summary, exercise recovery massage, were maintained manually. Booking logbooks and hundreds of patients’ files were stored at Wellness Centers. Since services at Wellness Centers are multiple in nature (gym, swimming pool, group classes, massage, spa area with sauna and steam rooms), and due to the unavailability of wellness receptionists, the integration of EHR has not been an easy step. Given the complexity of wellness-related functionalities, the new EHR system would require diverse involvement with supplementary tasks such as viewing referrals, appointment booking, rescheduling, cancellation and viewing patient’s laboratory, medications, or health records. The EHR system is designed to enable wellness providers to improve risk stratification, monitoring and management of exercise performance, lifestyle-related risk factors, and health conditions accordingly.

Evaluation of wellness providers’ preparedness in terms of knowledge and practical competencies towards EHR use, along with the provision of attentive training on functions and duties will optimize efficiency, timeliness, and completeness of health records. In February and August 2021, wellness providers underwent basic CIS (Clinical Information System) training and obtained access into EHR. However, due to the continued suspension of wellness services at RAK HC for more than two years, and staff being overwhelmed with COVID-19 duties, the current study was conducted as a quality improvement project to ensure the introduced EHR and practice meet expectations and overcome likely challenges especially for massage therapists taking the role of receptionists alongside their job.

Thus, the aim of this pre-post intervention study was (i) to evaluate the effectiveness of personalized EHR training on wellness providers’ knowledge and practical competencies, and (ii) to assess staff satisfaction regarding the EHR usage post-training. This study focuses on the evaluation of EHR training to solve the following questions: What is the level of improvement in the end users’ knowledge and competencies following 6 months of training, and what is the level of end users’ satisfaction relative to the received training.

## Materials and Methods

### Design, Setting and Participants

This study was conducted in the Wellness Center located at Rawdat-Alkhail Health Center, Primary Health Care Corporation (PHCC). It is sited in the central region of Qatar and was designated as a COVID-19 facility when the pandemic began. It is one of the six Wellness Centers across PHCC serving the adult population and offering a vast variety of interventions to empower people to adopt a healthier lifestyle such as gymnasium, swimming pool, and exercise recovery massage. Integration of EHR into wellness services was first established in August 2021 in all of the five Wellness Centers, with the exception of RAK which went live effectively in March 2022. The study population comprised RAK wellness staff whose job involves the usage of EHR including gym instructors and massage therapists/receptionists, both male and female (n = 14). Employees who are not involved in EHR or with prior knowledge and practice of EHR were excluded from the study (wellness supervisors, lifeguards, health coaches). A pre-post interventional study design was conducted between July 15, 2021, and March 1, 2022, to assess the impact of a 6-month training on knowledge, practical competencies and staff satisfaction related to the EHR system. Effectiveness on knowledge was measured as summative improvement in the number of respondents who were knowledgeable in different areas, while competencies were grounded in the improvement of skills specifically related to the job titles, functions, and duration of performance. Staff satisfaction relative to the training was measured no more than a week after the intervention.

### Sampling Technique

Due to the interventional nature of the study that was implemented in a solitary health center and to reduce sampling errors to achieve a desirable level of precision, all the individuals in the relevant population were recruited. We have included all male and female gym instructors (n = 10) and massage therapists/ receptionist (n = 4) working at RAK HC, Wellness Center, Doha, Qatar.

### Data Collection Instrument

The study instrument consisted of three sections, namely:

1. Section 1 elicited the sociodemographic of participants and self-reported EHR knowledge obtained via a semi-structured interview questionnaire. Knowledge on EHR system comprised a total of 12 items revealing the awareness, definition, areas of application, and advantages of EHR. A total score of less than 50% was considered as “poor overall knowledge” while 50% and above was considered as “good overall knowledge”.
2. Section 2 examined EHR practical competencies through 1:1 direct observation. Grounded on job titles, massage therapists/receptionists had to complete 12 competencies while 4 competencies for gym instructors, both of which related to functions including viewing, booking, and filling forms. Timeline of each competency was recorded in seconds to maintain timely system. A total score less than 50% was considered as “poor overall competency”, while 50% and above was considered as “good overall competency”.
3. Section 3 examined the staff satisfaction towards training and EHR use. After training, self-administered survey with 20 domains was distributed to the participants. Captured responses via a five-point Likert scale ranged from “strongly disagree” to “strongly agree”. All participants completed the questionnaire no more than a week following the training.

Sections 1 and 2 were obtained before and after the training intervention, while section 3 was only filled post-training.

### Intervention

**Figure 1:**
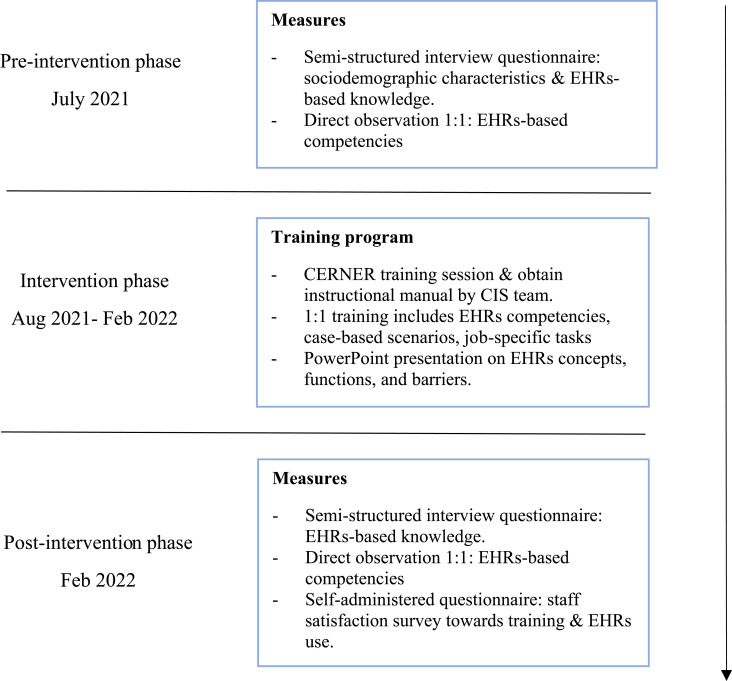
Overview of study phases and collected measures among participants.

Training was designed to engage wellness staff members in optimizing EHR potentials to support the efficient transfer from paper-based into EHR. Two trainers who were dedicated and credentialed to train staff on the EHR system delivered the training. The 6-month training curriculum was adapted to meet staff needs on specific job tasks that are based on their operational roles. Interventions involved CERNER training with provision of instructional manual by CIS team, staff rotation (one month each) at other operating Wellness Centers within external HCs (peer-learning and practice), case-based scenarios, assigning tasks including practice of booking and other CIS roles through 1:1 direct observation, and PowerPoint presentation about EHR. Frequent reporting of challenges and limitations of new EHR wellness package was continuously monitored throughout the study period. This study was conducted in three phases using a quantitative approach: pre-intervention, intervention, and post-intervention (Figure 1).

### Data Analysis

Frequency with percentages was calculated for categorical variables such as gender, job title, marital status etc. and mean with standard deviations for interval variables such as age in years, time in seconds and total mean percentage score of the wellness staff satisfaction questionnaire. Interquartile range (IQR) was also calculated for overall staff satisfaction. McNemar’s tests for pre-post intervention of categorical variables and paired Student t-tests for pre-post intervention of interval variables were applied wherever applicable. Strongly disagree, disagree, neutral, agree, and strongly agree were recoded as −2, −1, 0, +1, and +2 to make qualitative responses to quantitative responses. Mean percentage score of wellness staff satisfaction was calculated using the formula ∑ ((items response)/∑ (highest values in the items)) x100.^21^ P value 0.05 (two tailed) have been considered for statistical significance level. SPSS 28.0 statistical package has been used for the analysis.

## Results

### Participants

The mean age of participants was 38 ± 3.9 years out of which 71.4% were older than 35 years of age. Of the 14 participants, there were an equal number of male and female participants (50%). The majority of the participants (71.4%) were gym instructors, and the remainder were massage therapists/receptionists (28.6%). Of all respondents, (78.6%) were married, (14.3%) were single, and (7.1%) was a divorcee. All the participants (100%) have been working in Wellness Center for 5 years or more and were computer literate, 64.3% of which have indicated a previous experience in EHR use (Table 1).

**Table 1:** Sociodemographic Characteristics of Participants (n = 14)

Characteristics	Frequency	(%)
**Age (years)**		
< 35 years	4	28.6
≥35 years	10	71.4
Mean (+/- SD)	38±3.9	
**Gender**		
Male	7	50
Female	7	50
**Job title**		
Gym instructor	10	71.4
Massage therapist/receptionist	4	28.6
**Marital status**		
Single	2	14.3
Married	11	78.6
Divorced	1	7.1
Widow	-	
**Duration of practice at PHCC**		
< 5 years	0	0
≥ 5 years	14	100
Mean (+/- SD)	3.4±0.8	
**Have basic computer skills**	14	100
**Previous use of EHR**	9	64.3

**Abbreviations**: PHCC, Primary Health Care Corporation; SD, standard deviation; EHR, electronic health records.

### EHR Knowledge

Table 2 demonstrates knowledge of wellness staff towards EHR at pre- and post-intervention. All wellness staff (100%) had awareness of EHR at baseline. All respondents could correctly define EHR at post- compared with pre-intervention level (pre = 35.7% vs post =100%, p = 0.001). With regards to the areas of EHR applications, (100%) had knowledge about access to patients’ records at both levels. Most of the respondents had their knowledge improved in identifying other areas of applications including laboratory results (pre = 35.7% vs post = 78.6%, p = 0.03), treatment/drug management (pre = 42.9% vs post = 93.9%, p = 0.02), and data management and repository (pre = 35.7% vs post = 85.7%, p = 0.02) at post- compared with pre-intervention level. Most of the respondents had improvement in identifying the advantages of EHR at post- compared with pre-intervention level as the following: improve confidentiality of care (pre = 35.7% vs post = 100%, p = 0.001), reduce medical error (pre = 35.7% vs post = 85.7%, p = 0.02), improve quality of health care (pre = 35.7% vs post = 100%, p = 0.001), and reduce wait time (pre = 42.9% vs post = 85.7%, p = 0.03).

**Table 2:** Knowledge of Wellness Staff Towards EHR Pre and Post Intervention (n = 14)

Knowledge Component	Frequency	(%)	Frequency	(%)	P-value
	Pre-Intervention		Post-Intervention		
**Awareness of EHR**	14	100	14	100	-
**Definition of EHR**	5	35.7	14	100	0.001*
**Area of application of EHR**					
Patients’ record	14	100	14	100	-
Laboratory results	5	35.7	11	78.6	0.03*
Treatment/drug management	6	42.9	13	93.9	0.02*
Data management and repository	5	35.7	12	85.7	0.02*
**Advantages of EHR**					
Reduce workload	8	57.1	11	78.6	0.25
Improve confidentiality of care	5	35.7	14	100	0.001*
Reduce medical errors	5	35.7	12	85.7	0.02*
Improve quality of health care	5	35.7	14	100	0.001*
Reduce cost of health care	4	28.6	5	35.7	1.0
Reduce wait time	6	42.9	12	85.7	0.03*

**Note**: P-value ≤0.05.

**Abbreviation**: EHR, electronic health records.

### EHR Competencies

Table 3 represents the practical competencies of wellness staff to use the EHR system according to job category at pre-post intervention. A quarter (25%) of the massage therapists/receptionists could view and edit ambulatory organizer at pre- as compared with 100% at post-intervention level. All the massage therapists/receptionists (100%) were able to access PM (Person Management) office, select and access the patient chart, patient check-in and out, view/edit exercise recovery massage form, and view referrals at both pre- and post-intervention. However, the time (in seconds) had reduced substantially for viewing and editing ambulatory organizer (pre = 20±0 s vs post = 10±0 s), access to PM office (pre = 155±136 s vs post = 10±0 s), select and access patient chart (pre = 75±30 s vs post = 30±20 s), patients check-in and out (pre = 120±0 s vs post = 60±0 s), and view/edit exercise recovery massage form (pre = 135±75.5 s vs post = 60±0 s) from pre- to post-intervention level except for viewing referrals (pre = 90±35 s vs post = 90±35 s).

**Table 3:** Practical Competencies of Wellness Staff to Use the EHR System According to Job Category Pre-Post Intervention (n = 14)

Practical Competency	Frequency	(%)	Frequency	(%)
	Pre Intervention		Post-Intervention	
**A. Massage therapists/ receptionists (n = 4)**				
**Viewing & editing**				
**Ambulatory organizer**	1	25	4	100
Time (s) mean (+/- SD)	20±0		10±0	
**Access to PM office**	4	100	4	100
Time (s) mean (+/- SD)	155±136		10±0	
**Select & access to patient chart**	4	100	4	100
Time (se) mean (+/- SD)	75±30		30±20	
**Patients -check-in and out**	4	100	4	100
Time (se) mean (+/- SD)	120±0		60±0	
**View/edit Massage form**	4	100	4	100
Time (s) mean (+/- SD)	135±75.5		60±0	
**View referrals**	4	100	4	100
Time (s) mean (+/- SD)	90±35		90±35	
**Appointment booking and cancellation**				
**Book first gym assessment appointment**	4	100	4	100
Time (s) mean (+/- SD)	345±204		135±114	
**Book recurring appointments**	2	50	4	100
Time (s) mean (+/- SD)	330±42		255±90	
**Book for group class**	4	100	4	100
Time (s) mean (+/- SD)	300±155		150±104	
**Book massage appointment**	4	100	4	100
Time (secs) mean (+/- SD)	300±155		75±30	
**Cancel appointment**	4	100	4	100
Time (s) mean (+/- SD)	75±30		53±15	
**Reschedule appointment**	3	75	4	100
Time (s) mean (+/- SD)	80±35		53±15	
**B. Gym instructors (n = 10)**				
**Access ambulatory organizer**	4	40	10	100
Time (s) mean (+/- SD)	30±0		10±0	
**View/edit outcome gym summary form**	9	90	10	100
Time (s) mean (+/- SD)	101±57		71±36	
**View patients’ clinical data and labs**	9	90	10	100
Time (s) mean (+/- SD)	60±70		10±3	
**Place referral order**	5	50	10	100
Time (s) mean (+/- SD)	197±144		82±23	

**Abbreviations**: PM, person management; SD, standard deviation.

Also, all the massage therapists/receptionists could book first gym assessment appointments, recurring appointments, group classes, massage appointments, and cancel appointments at both pre- and post-intervention levels. However, the number had increased from 75% to 100% for rescheduling appointments. The time (in seconds) had also been reduced markedly for booking including first gym assessment appointments (pre = 345±204 s vs post = 135±114 s), recurring appointments (pre = 330±42 s vs post = 255±90 s), group classes (pre = 300±155 s vs post = 150±104 s), massage appointments (pre = 300±155 s vs post = 75±30 s), cancelling appointments (pre = 75±30 s vs post = 53±15 s), and rescheduling appointments (pre = 80±35 se vs post = 53±15 s) from pre-to post-intervention level.

On the contrary, all gym instructors could access ambulatory organizer (pre = 40% vs post = 100%), view/edit outcome gym summary form (pre = 90% vs post = 100%), view patients’ clinical data and labs (pre = 90% vs post = 100%), and place referral orders (pre = 50% vs post =1 00%) at post- when compared twith pre-intervention level. Similarly, the time had reduced to access the ambulatory organizer (pre = 30±0 s vs post = 10±0 s), view/edit the outcome gym summary form (pre = 101±57 s vs post = 71±36 s), view patients’ clinical data and labs (pre = 60±70 s vs post = 10±3 s), and place referral orders (pre = 197±144 s vs post = 82±23 s) from pre- to post-intervention level.

### Good EHR-Knowledge and Practical Competency Scores

When the EHR-knowledge and practical competency scores of wellness staff were analyzed, 100% of participants had achieved both good overall knowledge and practical competency scores at post-intervention as compared with 42.8% and 57.1% at pre-intervention, respectively (Table 4).

**Table 4:** Good Overall EHR-Knowledge and Practical Competencies Score of Wellness Staff Pre-Post Intervention (n = 14)

Good Overall Score	Frequency (%)	Frequency (%)
	Pre-Intervention	Post-Intervention
**EHR Knowledge**	6 (42.8)	14 (100)
**EHR practical competencies**	8 (57.1)	14 (100)

**Abbreviation**: EHR, electronic health records.

### Wellness Staff Satisfaction

**Figure 2:**
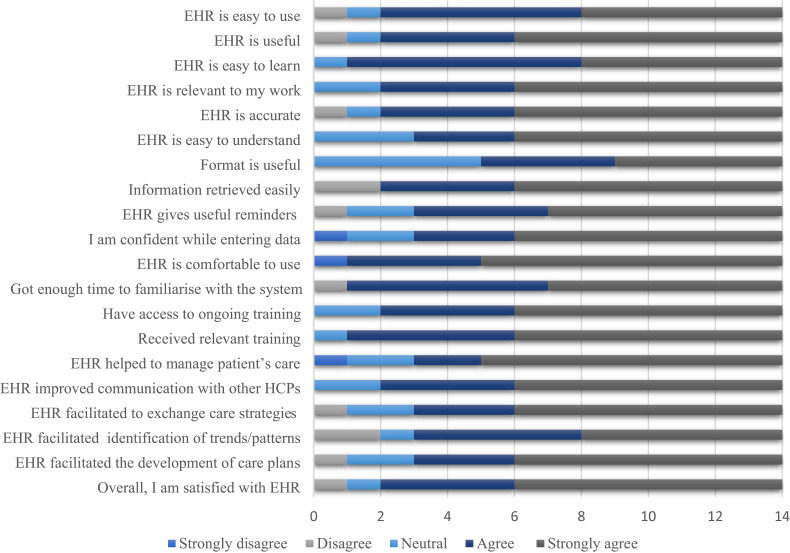
Wellness staff satisfaction towards EHR training.

Responses in the form of strongly disagree, disagree, neutral, agree, and strongly agree were collected for each item of the wellness staff satisfaction questionnaire. Strongly disagree, disagree, or neutral responses were much less for all the items in the questionnaire. Of the total of the 14 staff members, most of them agreed or strongly agreed for the 20 items of the questionnaire (Figure 2). The mean percentage score was 65.4±38.7 indicating a very good staff satisfaction towards EHR training.

## Discussion

This study was part of a quality improvement project conducted at a Wellness Center to evaluate the effectiveness of 6 months of tailored, one-to-one EHR training on the knowledge, practical competencies, and satisfaction of wellness staff with regard to the usage of a newly implemented EHR system.

Previous studies showed that amongst the factors critical to an EHR training program’s success was the use of hands-on exercises that were relevant to providers’ practice patterns.^15^,^16^ In a systematic review, Wiebe et al concluded that education was proved to be the most effective way to improve EHR documentation.^22^

In this study, the multi-approach intervention was successful in improving the perception of staff about the definition, areas of application, and advantages of EHR. This finding is consistent with previous work that identified health professionals who had good knowledge of an EHR system were about 2.12 times more likely to be ready to operate and accept the advantages of technology when compared with those with poor knowledge.^23^ Similarly, a pre-post study conducted at Tanta International Teaching Hospital, 2018 showed a statistically significant improvement in EHR knowledge and attitude (importance, function, components, and barriers) among nursing staff who received an educational program as compared with the control group.^24^ Numerous studies found that clinicians and staff consider lack of knowledge on EHR functions to be one of the challenges faced within using the system.^25^,^26^ In the present study, incorporating information related to EHR definition, reasons of implementation, and awareness within the training program were aligned with the concept of Coherence mechanism in the Normalization Process Theory (NPT) which emphasized that better knowledge and understanding of EHR reasons lead to better implementation of technology.^27^

Our study has also looked at the impact of training on practical competencies related to staff-specific job category and scope of practice. Significant changes after the intervention included: increased ability to view and edit patient records, improved booking/cancellation of appointments, place referral orders, and most importantly reduced the overall time required for EHR specific tasks. The current result concurs with several other studies which found that planned sessions of interactive and practical training brought about improvement in the confidence, preparedness, usage, and access to different EHR functions and tools among health-care providers.^28–30^ In contrary, DiAngi et al found that 20 months of an EHR training program has failed to decrease the self-reported or the calculated EHR use time, however, it improved the work satisfaction and the perception of work control.^28^ EHR use has also been linked to challenges associated with increased documentation time especially among health-care providers, raising a potential concern of efficiency and accuracy.^31^ Therefore, recording the duration of performing each task has been an integral component of this study, intending for optimum management and hence improvement in quality of care. In support of our study findings, a systematic review has revealed that documentation improvement indicators such as document accuracy, completeness, and timeliness of required tasks were observed following the EHR training.^22^

Furthermore, this study has illuminated a high degree of wellness staff satisfaction toward the EHR training. The findings are in keeping with results of other research which highlighted that adequate training is compatible with staff confidence while using EHR.^31^ Likewise, Devkota et al demonstrated that at least three weeks of EHR training improved the quality and efficiency of care delivery owing to staff positive cognition, attitude, and satisfaction.^32^ Multiple studies provided evidence for the association between EHR use and provider burnout for several reasons involving time requirement, clerical burden, and distraction from patient’s care.^33^,^34^

In accordance with our findings, Lanier et al in their pre-post intervention study found that participants who received 3 months of training tended to feel more comfortable with the EHR use and less likely to regard it as a barrier to successful patient-provider relationship.^33^ Makam et al found that providers’ dissatisfaction was noted especially in documentation of preventative services and chronic disease management.^35^ The authors noted that optimizing provider use of key functions of EHR was a priority.^35^ Additionally, in Saudi Arabia, Al-Otaybi et al reported temporary loss of access to patient records in the event of a computer crash or failed power, followed by privacy and security concerns, as the main EHR barriers leading to staff dissatisfaction.^36^

Implementation of EHR requires continuous auditing and quality improvement. Meehan et al found that most of the primary practices were not ready to bring about improvement in the processes or outcomes of care as they did not have the required quality improvement knowledge or skill.^37^ The most common barriers were inadequate number of supportive staff and insufficient knowledge and skill of quality improvement capabilities and functionality.^37^

## Limitations

Although we have delivered a multi-component intervention to train and ensure efficiency of wellness staff through our study, several challenges exist. First, the small study sample and the convenience recruitment method, which was undertaken across a single Wellness Center, could have led to sampling bias as well as threaten the generalizability of our findings. To overcome selection bias, we recruited all gym instructors and massage therapists/receptionists working in the study setting. The promising effect of the tested training may be considered transferable to other Wellness Centers. Additionally, due to the small number of participants, we could not perform advanced statistics to understand the demographic profile in relation to outcomes of interest.

Second, the study has measured short-term outcomes prior to reopening of the Wellness Center in RAK HC, however, actual performance, impact on patient care, as well as retaining of knowledge and competencies related to EHR were not considered. To minimize recall bias and correctly measure the immediate effectiveness of the delivered training, staff satisfaction survey was completed no more than a week following the training.

Third, the fact that wellness supervisors were the ones who conducted part of the training, may have given rise to respondent bias. However, we ensured standardized protocols in place for data collection such as developing a training template with learning objectives, required timeline/hours of exposure as well as training of the interventionists to reduce inter-observer variability and interviewer bias.

Finally, although our study compares changes between matched pairs, we did not have a control group which may affect the validity of the results and some of our improvements could be attributed to self-improved use of the EHR over time regardless of the training. Being involved in COVID-19 related duties (many of which were computer-based) at similar time of intervention, it is possible that some unknown confounders influenced the knowledge and practical skills and consequently explain the improved levels of such variables.

## Implication on Clinical Practice & Research

Outcomes of this study will be applicable to health-care and other organizations, quality improvement specialists, policymakers and will provide a direction for future researchers seeking to improve patient safety through completeness and timeliness of EHR tasks.

Our work serves as an initial foundation for the creation of a significant investment in a tailored EHR training. It is an opportunity for health-care organizations and departments to streamline, standardize, and improve the care process. Increasing awareness, knowledge, and skills of the end users on EHR before system implementation is necessary to maximize its potential. Identifying the areas and requirements prior to the implementation will help to improve self-efficacy, system and information quality, productivity, and user/patient satisfaction.

The results of the study showed that optimizing EHR skills via training has resulted in reducing time interfacing with all aspects of functionalities and patients’ health records. User efficiency was translated to a large extent into system efficiency through outputs such as waiting time or consultation time, therefore better patient care. Given these results, we advocate for health-care organizations to increase the EHR education and support their providers to improve patient care and promote staff satisfaction as a result. We also advocate for these organizations to adopt targeted, functions-centered training as a core competency for any new joiners.

The findings from this quality improvement project provide formative evidence that personalized, intensive training effectively improved end user knowledge and practical skills related to EHR use prior to actual implementation. This training approach could also be adapted for use in other settings and serves a different purpose for different stakeholders. Leaders should consider continuous monitoring and auditing to ensure the sustainability of competencies.

Based on the results of this study, the authors propose several important health policy matters that should be considered to promote best practice and successful adoption of EHR. Policymakers may use the results as objective evidence to plan, prioritize interventions, and allocate resources accordingly. Finally, we anticipate that these findings will serve as the groundwork for future studies on EHR end user preparedness for change. It would be of interest to analyse which factors related to staff and patient might have an influence on the readiness for EHR usage.

Research that measures preparedness for change and correlated factors should be prioritized to provide a holistic EHR pre-implementation assessment. Longitudinal studies are warranted to further measure sustainability of improvement as well as long-term outcomes on care delivery such as adherence and completeness of a 12-week wellness program, cardiopulmonary fitness, and biomedical measures.

## Conclusion

This preliminary study provides evidence that targeted, multi-approach, and function-centered training has effectively improved wellness staff knowledge and competencies in EHR-related functionalities. This will be translated to improvement in quality, accuracy, timeliness, and safety of the provided health care. Enhancing staff critical skills and reducing time interfacing with all aspects of health records will reduce staff burnout and promote patient satisfaction. Continuous monitoring remains the key for evaluating and achieving long-term system efficiency.

## Data Availability

Data are available from the author (S.M.) upon reasonable request.
